# Serine Protease HTRA1 Membranous Nephropathy With Polytypic IgG Concurrent With Plasma Cell Dyscrasia

**DOI:** 10.1016/j.xkme.2026.101402

**Published:** 2026-05-12

**Authors:** Jonathan E. Zuckerman, Sarah Larson, Lama Abdelnour

**Affiliations:** 1 Department of Pathology and Lab Medicine, David Geffen School of Medicine at the University of California Los Angeles, Los Angeles, CA; 2 Division of Hematology Oncology, David Geffen School of Medicine at the University of California Los Angeles, Los Angeles, CA; 3 Division of Nephrology, David Geffen School of Medicine at the University of California Los Angeles, Los Angeles, CA

**Keywords:** Membranous nephropathy, nephropathology, renal pathology, HTRA1, multiple myeloma, plasma cell neoplasm, plasma cell dyscrasia HTRA1-associated membranous nephropathy, progressive proteinuria, paraneoplastic membranous nephropathy, polytypic IgG, IgA-lambda multiple myeloma

## Abstract

A growing number of target antigens have been identified in membranous nephropathy (MN) in recent years. Clinical correlations exist for some MN antigens, whereas others remain poorly characterized because of their rarity. High-temperature requirement A serine peptidase 1 (HTRA1) is the target antigen in approximately 1%-2% of MN cases without any established disease associations. Recent studies suggest HTRA1-MN may associate with malignancies in approximately 12% of cases, mostly solid tumors. To date, only 2 cases of HTRA1-MN have been reported in the setting of atypical hematopoietic disorders, including chronic lymphocytic lymphoma and monoclonal gammopathy of uncertain significance. Here, we present a case of HTRA1-MN with polytypic IgG deposits in a 62-year-old man with a plasma cell dyscrasia (IgA monoclonal gammopathy and 20% bone marrow involvement by a λ-restricted plasma cell neoplasm) who presented with nephrotic syndrome. A limited initial kidney biopsy suggested early MN. Daratumumab-based induction therapy resulted in a partial renal response (urinary protein-creatinine ratio of 0.7 g/g). However, proteinuria subsequently recurred (∼6.7 g/g), prompting a repeat biopsy that demonstrated HTRA1-MN with polytypic IgG staining. Proteinuria has progressively worsened (12 g/g), with persistent minimal residual plasma cell disease despite ongoing daratumumab maintenance therapy. To our knowledge, this case represents only the second reported case of HTRA1-MN occurring in the setting of monoclonal gammopathy, the first in multiple myeloma, and a rare example of a nonmonotypic MN in this context. Temporal proximity and partial response to anti-plasma cell therapy suggest a possible paraneoplastic relationship, although a causal relationship remains unproven.

Several newly identified target antigens in membranous nephropathy (MN) have emerged in recent years.^1^ For some, such as NELL-1 and FAT-1, clear clinical correlations with underlying diseases or environmental exposures have been established. In contrast, potential secondary disease associations with other antigens remain uncertain and are difficult to evaluate because of the small number of reported cases.

High-temperature requirement A serine peptidase 1 (HTRA1) is the target antigen in 1%-2% of MN overall (∼4% of PLA2R-negative cases), and no clear disease associations have been established. However, emerging data indicate a malignancy association in approximately 12% of cases, mainly solid tumors (eg, prostate, lung), with one case reported in chronic lymphocytic leukemia (CLL) and a second in a patient with monoclonal gammopathy.^2^^,^^3^ Here, we present a case of HTRA1-MN with polytypic IgG deposits occurring in a patient with multiple myeloma.

## Case Report

A 62-year-old man presented with chief complaints of 1-month of foamy urine, new-onset of peripheral edema, and abdominal distention. His medical history included a remote excised dermal melanoma and gout. His serum creatinine level was 1.19 mg/dL, and serum albumin level was 2.0 g/dL. Urinalysis showed 2+ blood and 3+ protein, with a urinary protein-creatinine ratio (UPCR) of 8.5 (a urinalysis performed 1.5 years earlier showed no proteinuria or hematuria).

Serologic evaluation demonstrated a monoclonal IgA-λ gammopathy in serum and urine. Antinuclear antibody, antineutrophil cytoplasmic antibody, anti-PLA2R antibody, and hepatitis serologies were negative. Bone marrow biopsy demonstrated a λ-restricted plasma cell neoplasm involving 20% of marrow cellularity. Fluorescence in situ hybridization studies on CD138-positive cells demonstrated monosomy 13, IGH gene rearrangement, and 3’IGH deletion or unbalanced IGH rearrangement. The patient had no other systemic manifestations of his plasma cell dyscrasia.

A kidney biopsy (not pictured) was performed, which demonstrated no significant light microscopic changes in glomeruli and stage I subepithelial deposits using electron microscopy. The biopsy was limited with no glomeruli in the conventional immunofluorescence tissue and insufficient glomeruli for salvage immunofluorescence studies or membranous antigen testing. No other definite paraprotein-related glomerular or tubulointerstitial lesions were identified. Early MN was diagnosed, with secondary etiologies (beyond the plasma cell dyscrasia) excluded clinically.

The patient was treated with 6 cycles of daratumumab, lenalidomide, bortezomib, and dexamethasone, followed by maintenance monthly daratumumab. A subsequent bone marrow biopsy demonstrated minimal residual disease (1,192 residual clonal cells per million nucleated cells). Proteinuria and edema improved, with a UPCR of 0.7 at the end of cycle 6. However, after completing the initial induction phase therapy, the patient’s proteinuria worsened to 6.7 g/g approximately 2 months post-therapy, prompting a repeat kidney biopsy approximately 6 months after the initial biopsy and approximately 1 week after the second bone marrow biopsy noted above.

**Figure 1:**
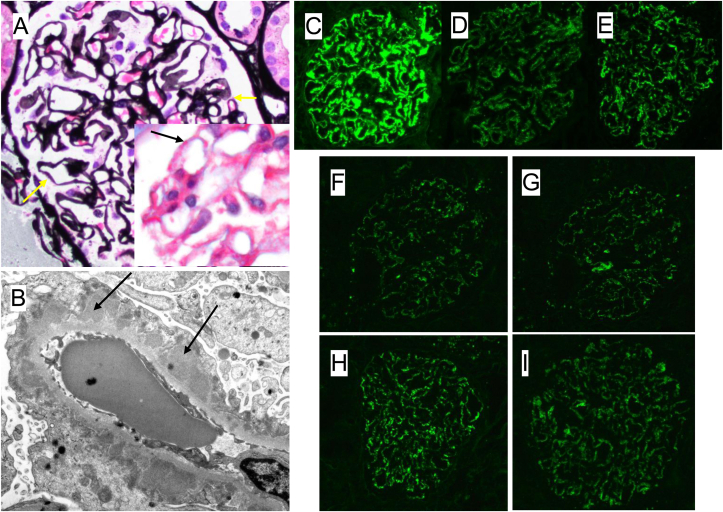
Kidney biopsy morphology. (A) Jones' silver stain showing a near-normal glomerulus with subtle spikes and intramembranous lucencies (arrows) consistent with early MN (original magnification 400x). (A, inset) Masson trichrome stain showing small granular subepithelial deposits (×600). (B) Electron micrograph showing numerous subepithelial deposits (arrows) and spike formation compatible with stage 2 MN (×9300). Immunofluorescence demonstrates diffuse granular predominantly capillary wall staining for (C) IgG, (D) κ, (E) λ, (F) IgG1, (G) IgG2, (H) IgG3, and (I) IgG4, consistent with polytypic IgG deposition (fluorescein isothiocyanate channel, ×400 for all).

The second kidney biopsy (Fig 1) contained renal cortex with 38 glomeruli (6 globally sclerotic). Light microscopy showed rare segmental intramembranous lucencies and vague spikes in glomerular capillary walls on silver stain and small subepithelial deposits on the trichrome stain. There was no significant mesangial or endocapillary hypercellularity. There was no segmental sclerosis, glomerular necrosis, or crescent formation. There was <5% interstitial fibrosis and tubular atrophy. There were no atypical casts. Congo red stain was negative. Immunofluorescence demonstrated diffuse global granular capillary wall staining with IgG (3+), C1q (trace), C3 (2-3+), κ light chain (2-3+), and lambda light chains (3+). Very focal granular tubular basement membrane (TBM) deposits were positive for IgG (2+), κ light chain (trace), and lambda light chain (1+). The IgA immunofluorescence stain was negative in glomeruli.

IgG heavy chain subclass staining demonstrated diffuse global granular capillary wall staining with IgG1 (1+), IgG2 (1-2+), IgG3 (2+), and IgG4 (1+) (TBM deposits were not detected in these sections). Immunofluorescence following pronase digestion for IgA heavy chain was negative for masked IgA deposits. Electron microscopy showed global, finely granular subepithelial electron-dense deposits with early capillary spike formation, and rare small mesangial deposits. TBM deposits were not present. There were no tubuloreticular inclusions, powdery, crystalline, fibrillary, or other substructured deposits present. PLA2R immunostain was negative.

Liquid chromatography–tandem mass spectrometry performed on microdissected glomeruli identified a peptide profile consistent with serine protease HTRA1. A diagnosis of HTRA1-associated MN was rendered.

Treatment with daratumumab, carfilzomib, and dexamethasone was planned to obtain a deeper response; however, the patient could not tolerate carfilzomib. Subsequent therapy with rituximab was initiated in addition to maintenance daratumumab. The patient’s proteinuria has gradually worsened over time since the second kidney biopsy. The UPCR 3 months later increased to 12.3 g/g (∼ 1 month after the first rituximab infusion). Flow cytometry from a bone marrow aspirate 1.5 months after the secondary kidney biopsy was negative for monotypic plasma cells and fluorescence in-situ hybridization studies on the aspirate demonstrated at least a small population of plasma cells with the IGH gene abnormalities as above. The IgA-λ paraprotein remains detectable in the patient serum (0.3 g/dL) at the time of most recent proteinuria assessment.

## Discussion

Only a small number of HTRA1-MN are described in the literature. This antigen was first described by Al-Rabadi et al,^4^ who studied a large cohort of “quadruple negative” (PLA2R, THSD7A, NELL-1, and EXT2-negative) MN cases and identified HTRA1 as the target antigen in 14 cases (approximately 1% to 2% of all MN cases). Patients were typically older than in other forms of primary MN (mean age 67.3 years), with a male-to-female ratio of 4:3, and a predilection for White patients. Most cases were IgG4 predominant, and atypical membranous features, such as segmental immune complex deposition and mesangial deposits, were seen in a subset. Circulating anti-HTRA1 antibodies correlated with disease activity. Tubular basement membrane deposits were not described, and no clear secondary causes (eg, malignancy, infections, or systemic autoimmune diseases) were identified. One case had stage IV small-cell lung cancer, which preceded the MN by 2 years, without proteinuria at cancer diagnosis.

Since that initial report, only rare cases of HTRA1-MN have been published without consistent disease associations. A recent case described HTRA1-MN in CLL^2^ with MN improvement postchemotherapy. However, the MN occurred 19 years after initial CLL diagnosis. Although the MN improved following CLL-directed therapy (rituximab, cyclophosphamide, and prednisone), such treatments would also likely be effective for primary MN. Recent cohort data showed malignancies in 12% of HTRA1-MN cases limited to solid tumors.^3^ There is one report of a HTRA1-MN occurring in a patient with an IgG-κ monoclonal gammopathy of uncertain clinical significance without response to bortezomib treatment.^5^ No prior cases of clear plasma cell neoplasm with monotypic plasma cells demonstrated in a bone marrow biopsy have been described.

MN is a well-recognized paraneoplastic phenomenon in the setting of solid organ malignancies.^6^ Other hematologic malignancies, including CLL and non-Hodgkin lymphomas, have also rarely been associated with MNs.^7^, ^8^, ^9^ At least a subset of monotypic MN cases have been documented concurrent with CLL, and monoclonal MN is currently considered a lesion of monoclonal gammopathy of renal significance in the absence of overt hematopoietic malignancy.^10^^,^^11^

MN in the setting of multiple myeloma, and plasma cell dyscrasia more broadly, is rare.^12^ Notably, most reported cases of MN in multiple myeloma lack detailed immunofluorescence descriptions, particularly regarding monotypic versus polytypic deposits, which would clarify whether the MN represents a true paraprotein-related process. Interestingly, one such reported case occurred with a biclonal IgA gammopathy.^12^

In the present case, monotypic IgG deposition was excluded, as immunofluorescence staining was positive for both κ and λ light chains as well as all 4 IgG subclasses. The close temporal relationship between MN onset and myeloma diagnosis, the early stage of the MN, minimal chronic changes on biopsy, partial initial response to myeloma-directed therapy, and subsequent progressive proteinuria in the setting of ongoing minimal residual disease all support the possibility of an association. However, a causal relationship between the MN and the patient’s plasma cell dyscrasia is uncertain; if related, the MN may represent a paraneoplastic mechanism similar to MN observed with solid tumors.

This is the second reported case of HTRA1-MN occurring in the setting of plasma cell dyscrasia or monoclonal gammopathy, the first in the setting of multiple myeloma, and a rare example of a nonmonotypic MN in this context overall. Given the rarity of HTRA1-associated MN, further recognition of such exceptional cases may help clarify potential secondary disease associations.
